# The Effects of Lack of Joint Goal Planning on Divorce over 10 Years

**DOI:** 10.1371/journal.pone.0163543

**Published:** 2016-09-26

**Authors:** Judith Gere, David M. Almeida, Lynn M. Martire

**Affiliations:** 1 Department of Psychological Sciences, Kent State University, Kent, Ohio, United States of America; 2 Center for Healthy Aging, Pennsylvania State University, University Park, Pennsylvania, United States of America; University of Toledo, UNITED STATES

## Abstract

Given the negative consequences of divorce on health and well-being, it is important to try to identify its predictors. In the current study we used data from the National Survey of Midlife Development (*N* = 2801) to examine the longitudinal effects of lack of joint goal planning with a romantic relationship partner on divorce over a 10-year period. Multilevel regression analyses showed that lack of joint planning with the relationship partner was associated with a 19% increase in the odds of divorce, even when controlling for various demographic (i.e., age, gender, relationship length, number of children in the household), individual (i.e., neuroticism, positive affect, negative affect, physical symptoms, planning), and relationship (i.e., marital empathy, partner strain, partner disagreement, marital satisfaction, commitment). These results demonstrate the importance of considering one’s partner when making decisions and plans for the future, given that it has clear implications for relationship dissolution.

## Background

Romantic relationships, arguably the most intimate relationships for most people, are one of the strongest predictors of overall happiness [1]. People in high quality romantic relationships also enjoy better mental and physical health [2–4]. Unfortunately, many romantic relationships do not last a lifetime. In the United States, 40% of first marriages end in divorce [5], and when relationships go awry, they are a source of considerable distress. People in poor quality intimate relationships have worse health than those in good quality relationships or no romantic relationship [3–4]. Moreover, relationship dissolution can result in long-term decreases in well-being [6], interfere with healthy biological processes, leading to an increased risk of physical health problems [4, 7], and lead to the onset or worsening of mental health problems [4]. A meta-analysis has shown that divorce is also associated with increased risk for early death [8].

Given the negative consequences of relationship dissolution, it is important to identify its predictors. A better understanding of factors that predict relationship dissolution might make it possible to mitigate their effects on the relationship or even prevent the decline in marital quality that often precedes divorce through targeted interventions. Much of the existing literature on predictors of divorce has been focused on demographic (e.g., number of children, relationship length) or individual factors (e.g., neuroticism). However, relationship-specific predictors, such as negative relationship behaviors, are also important predictors of divorce (e.g., [9,10]). In the current study, we focus on the role of lack of joint goal planning with a romantic relationship partner as an important factor in predicting relationship dissolution.

## The Importance of Joint Planning

According to interdependence theory, when romantic partners live together, their lives become closely intertwined, such that the decisions made by one partner have a direct impact on the other partner [11–13]. Such high levels of interdependence present many challenges for the partners because their decisions impact not only their own lives and outcomes, but also those of their partner, and no two people have goals that are perfectly aligned [12–15].When the decisions and activities of one partner impact the other partner, it becomes very important for them to coordinate their actions effectively, to ensure both that they are able to meet their own needs and wants, and that the desires of their partner are also taken into account.

Effective coordination between partners’ actions is important for several reasons. Inefficient coordination has been shown to tax people’s self-regulatory resources [14,16]. This is problematic because self-regulation is important for enacting pro-relationship behaviors that enable people to be responsive to their partner’s needs, especially when those are not in line with their own desires [17–18]. As such, when partners are not able to coordinate their actions effectively, it may be more difficult for them to focus on the relationship and each other. Self-regulatory resources are also needed for making progress towards goals, that is, towards people’s desired end-states [19]. Being able to meet one’s own needs and desires, which occurs through the pursuit of personally meaningful goals, is an important ingredient of high levels of well-being [20–22]. The ability to continue to meet one’s own needs and make personal goal progress in the context of a romantic relationship is therefore important to ensure the viability of the relationship and the well-being of the partners [23–25].

Furthermore, if partners choose not to coordinate their actions with each other and instead make their decisions and plans without regard to their relationship, it is likely that they would encounter many problems given their levels of interdependence. Disregard for the other person when one’s decisions impact the outcomes of the other is likely to build resentment and lead to conflict between the partners [12,13,18,26]. When the decisions made by one partner interfere with the other partner’s plans, the result may not only be conflict between the partners but also difficulty for the other partner to carry out their own plans and make progress toward their own goals. Thus, when the lack of coordination is purposeful and intentional, the long term effects may be continuing interference with each other’s actions and ineffective coordination of actions. Such lack of coordination may lead to conflicts between partners and declines in well-being. Over time, lack of coordination could potentially lead to relationship dissolution.

Given both the difficulty and importance of coordinating relationship partners’ activities, it is important to identify factors that may enable partners to more effectively coordinate their actions. Both partners need to ensure that their own desires are taken into account and they are able to pursue their personal goals in the context of their relationship. People pursue multiple goals, as they have many wants and needs, some of which are long-term (e.g., become more physically fit) while others are more short-term (e.g., see a movie Friday night). Individuals move towards their desired end-states through making both long-term plans and short-term day-to-day decisions [19]. Thus, when people try to effectively coordinate their actions with their partner, it is important that the coordination occurs both at the level of daily decisions (e.g., do I need to head home and pick up dinner on the way or can I go to the gym after work?) and long-term plans (e.g., should I take a job in another city?).

One factor that may enable partners to coordinate their actions more effectively is by making sure that they keep each other informed about their plans and decisions. In addition, seeking the partner’s input on plans and decisions would enable the partners to better coordinate their actions in a way that is more likely to meet the needs of both partners. In the context of a romantic relationship, people cannot always do what they want, as at times they have to prioritize the relationship or the needs of their partner if they want the relationship to continue [12,13,17,18,26]. Openly communicating with the partner and discussing one’s plans and decisions regarding things that also impact the partner ensures that one is aware of the partner’s preferences and can prioritize them for the sake of the relationship when necessary. Thus, we believe that making decisions and planning together with the partner would enable relationship partners to coordinate their actions more effectively and result in greater ability for both partners to make progress towards their own goals and to get their own needs met within their relationship, making relationship dissolution less likely.

Furthermore, involving the relationship partner in decision making and planning for the future may also result in the partner feeling included and valued in one’s life. Such feelings may then lead to higher supportiveness from the partner, which would then enable people to more effectively carry out their plans [27–28]. In turn, the partner’s supportiveness and people’s greater ability to move toward their own goals is associated with higher individual and relational well-being [29], which serve to further reduce the odds of relationship dissolution.

## The Current Study

In the current study, we examined whether people who make less effort to plan and make decisions jointly with their cohabiting romantic relationship partner are at a higher risk of relationship dissolution over time. Thus, we tested whether lack of joint planning prospectively predicts increased odds for divorce over a 10-year period. Given the importance of effective action coordination between romantic partners, we wanted to examine whether a lack of joint planning predicts divorce above and beyond various demographic, individual, and other relationship factors that would also be expected to be associated with higher odds of divorce and/or lack of joint planning. We expected that lack of joint goal planning would predict divorce even when known predictors are accounted for, indicating that joint planning has independent effects on divorce and can contribute to its prediction.

In terms of demographic factors, we examined the association between lack of joint planning and divorce independent of age, relationship length, and number of children. Prior research has shown that individuals who are older and are in more stable relationships (i.e., relationships that have lasted longer) are less likely to divorce [2,9]. Furthermore, those who have children are also less likely to divorce [9]. We also included gender in our analysis as a demographic covariate although we did not expect it to be associated with divorce, because we also wanted to examine its potential interactive effects with lack of joint planning in predicting divorce.

Regarding individual factors, we controlled for neuroticism, negative affect, positive affect, physical symptoms, and general tendency to plan. Neuroticism and negative affectivity are both well-known predictors of divorce [30–32], and positive affect has been shown to predict marital stability [2,31]. We controlled for physical symptoms because poor physical health is a known stressor that impacts the marital relationship [2,9]. Furthermore, we also included a measure of people’s general tendency to make plans to ensure that the effects of our primary variable of interest, lack of joint planning, are not due to a general likelihood of making plans but rather, the effects are specifically due to lack of joint planning with the relationship partner.

Finally, given that marital quality is an important predictor of divorce (e.g., [9,10], we wanted to control for various indicators of marital quality. We controlled for partner strain and marital satisfaction, as strain indexed the general level of tension between the partners and satisfaction indexed people’s global evaluations of their relationship. We wanted to examine whether lack of joint planning predicts divorce over and above negativity already present in the marriage at Time 1. Similarly, commitment predicts marital stability beyond the quality of the marriage (e.g., [33–34]), thus we assessed and controlled for relationship commitment. We also controlled for martial empathy because we wanted to examine whether lack of joint planning is associated with divorce even when the partner’s general responsiveness to one’s needs is taken into account. Furthermore, we assessed partners’ disagreement on various issues to determine if the effects of lack of joint planning are accounted for by the partners’ general levels of consensus when they make plans or decisions together.

In sum, we wanted to test whether lack of joint planning is predictive of divorce across time, even when other factors that are known to predict divorce are taken into account. To test our predictions, we used a national longitudinal data set that includes assessments of joint planning and other demographic, individual, and relationship variables at the initial time point, and assessed people again 10 years later.

## Method

### Participants and Procedures

We used data from the National Survey of Midlife Development (MIDUS), a national two-wave panel study of English-speaking adults between 25 and 74 years of age in the United States. The first wave of data collection took place 1995–1996, and the second wave took place 2004–2006. Data were collected via telephone interviews and self-administered questionnaires. In the current study, we used data only from the self-administered questionnaires, as the relevant constructs were assessed in that component of the MIDUS. The MIDUS sample (*N* = 7108) consists of four subsamples: a national random digit dialing sample, siblings of individuals in the random-digit sample, oversamples from five metropolitan areas, and a national random digit dialing sample of twins. 70% of participants (75% adjusted for mortality; *N* = 4963) also provided data at T2. Given that we used data from an already existing data set, we were not involved in determining sample size and did not make recruitment and data-collection stopping decisions. The data set is publically available in de-identified and anonymized form and was downloaded from the MIDUS website for analysis.

Given our interest in the outcome of relationship dissolution, we only used data from participants who indicated at T1 that they were either married or living together with a relationship partner (*N* = 3481), and for whom we were able to determine whether they were in the same relationship at T2. We classified people as still in the same relationship if they listed the same year that their relationship began (e.g., year of current marriage) at both time points. We classified people as no longer in the same relationship if at T2 they indicated that 1) they were no longer living in a marriage-like relationship with a partner, 2) they were divorced or separated, or 3) they indicated a year of current marriage that differed from the year of current marriage reported at T1 and/or that fell into the time period between the two data collection sessions. Participants who became widowed were excluded from analyses.

In total, we were able to determine the relationship status of 2801 (80.5%) out of 3481 individuals who were in cohabiting or marital relationships at T1. At T2, 2447 (87.4%) were still with the same relationship partner and 354 (12.6%) were no longer with their T1 partner and have experienced the dissolution of their relationship at some point during the 10-year period. At T1, the mean age of our sample was 46.2 years (*SD* = 11.9, range 24 to 74) and men and women were equally represented (49.0% men, 51.0% women). A total of 98.7% of participants were married and the remaining 1.3% were living with a partner in a steady, marriage-like relationship (given that the majority of the sample were married, we refer to relationship dissolution as divorce throughout the paper). Participants indicated that they were together with their current partner for an average of 21.6 years (*SD* = 13.3, range 0 to 58 years), and had 2.4 children in the household on average (*SD* = 1.46, range 0 to 10).

### Measures

#### Planning without the partner

Participants’ lack of planning together with the partner was measured at T1 using three items (i.e., “My partner and I are a team when it comes to making decisions,” “I don’t make plans for the future without talking it over with my partner,” and “When I have to make decisions about medical, financial, or family issues, I ask my partner for advice”). Items were rated on 7-point scales (1 = strongly agree to 7 = strongly disagree) and were averaged for analysis (*M* = 1.73, *SD* = 1.01, α = .82). Higher scores indicated higher levels of planning without the partner. Only 37% of individuals were at the lowest level of lack of joint planning. The remaining 63% of individuals’ scores were distributed along the full scale, with decreasing frequencies across the scale, as lack of joint planning increased.

#### Individual psychosocial variables

At T1, participants provided information with regards to several psychosocial variables that we used as covariates in our analyses. They rated their experiences of *positive affect* (cheerful; in good spirits; extremely happy; calm and peaceful) and *negative affect* (so sad nothing could cheer you up; nervous; restless or fidgety; hopeless) over the past 30 days [35]. Items were rated on 5-point scales (1 = all of the time to 5 = none of the time), and were coded such that higher scores represented higher positive (*M* = 3.45, *SD* = .65, α = .86) and negative affect (*M* = 1.51, *SD* = .59, α = .80), and each scale was averaged for analysis.

Participants indicated their experiences of seven *physical symptoms* (e.g., headaches, trouble sleeping, irritability) over the past 30 days on 6-point scales (1 = almost every day to 6 = not at all). Items were reverse coded, such that higher scores indicated more frequent physical symptoms, and were averaged for analysis (*M* = 2.19, *SD* = .89, α = .69).

In order to measure participants’ level of *neuroticism*, they indicated to what degree each of four adjectives (moody, worrying, nervous, calm) described them on 4-points scales (1 = a lot to 4 = not at all) [36]. Items were coded such that higher scores indicated higher levels of neuroticism, and were averaged for analysis (*M* = 2.20, *SD* = .66, α = .76).

Participants also completed a measure to assess their *tendency to make plans* in general. They rated the extent to which each of three statements were true of them (i.e., “I like to make plans for the future,” “I know what I want out of life,” and “I find it helpful to set goals for the near future”) on 4-point scales (1 = a lot to 4 = not at all) [37]. Items were coded such that higher scores indicated higher planning, and were averaged for analysis (*M* = 3.19, *SD* = .63, α = .73).

#### Relationship quality variables

At T1, participants rated several aspects of the quality of their relationship. *Marital empathy* was measured using six items (e.g., “How much does your partner understand the way you feel about things”), which were rated on 4-point scales (1 = a lot to 4 = not at all) [38–39]. Items were reverse scored, such that higher scores indicated higher marital empathy, and were averaged for analysis (*M* = 3.61, *SD* = .53, α = .90).

*Partner strain* was measured using six items (e.g., “How often does your partner make you feel tense”), which were rated on 4-point scales (1 = often to 4 = never) [38–39]. Items were reverse scored, such that higher scores indicated higher partner strain, and were averaged for analysis (*M* = 2.21, *SD* = .59, α = .87).

*Partner disagreement* was measured with regards to three areas of disagreement (money matters, household tasks, leisure activities) that were rated on 4-point scales (1 = a lot to 4 = not at all) [40]. Items were reverse scored, such that higher scores indicated higher levels of disagreement, and were averaged for analysis (*M* = 2.05, *SD* = .71, α = .71).

*Marital satisfaction* was measured using one item (“How would you rate your marriage or close relationship these days”), which was rated on an 11-point scale (0 = worst to 10 = best) with higher scores indicating higher satisfaction (*M* = 8.25, *SD* = 1.78).

*Commitment* to the relationship was assessed using one item (“Realistically, what do you think the chances are that you and your partner will eventually separate?”), which was rated on a 4-point scale (1 = very likely to 4 = not likely at all). Higher scores indicated higher levels of commitment to the relationship (*M* = 3.55, *SD* = .68).

## Results

### Preliminary Analyses

Table 1 presents the zero-order correlations between all of the variables used in the analyses. As expected, most of the variables were significantly associated with divorce. We also conducted a series of logistic regressions, using each control variable as a sole predictor of divorce. All of the control variables were significant predictors of divorce, except for gender, physical symptoms, and general tendency to plan. These results underscore the importance of many of these variables in the prediction of divorce. However, some of these variables were highly correlated with one another (e.g., age and relationship length, see Table 1). Given that multilevel regression analysis examines unique prediction and eliminates the shared variance between constructs, we expected that many of these control variables would not reach statistical significance in our models with multiple predictors. Our primary goal in this paper was to test the hypothesis that even when shared variance between these constructs and lack of joint planning is eliminated, lack of joint planning will still add to the prediction of divorce and emerge as a significant unique predictor.

**Table 1:** Zero-order correlations between the variables.

	1	2	3	4	5	6	7	8	9	10	11	12	13	14	15
1. Divorce															
2. Lack of joint planning	.16*														
3. Age	-.20*	-.05*													
4. Gender	.02	.01	-.10*												
5. Relationship length	-.22*	-.03	.81*	-.02											
6. Number of children	-.08*	.05*	.38*	.01	.30*										
7. Neuroticism	.07*	.14*	-.14*	.13	-.09*	-.06*									
8. Positive affect	-.07*	-.22*	.09*	-.02	.07*	.01	-.47*								
9. Negative affect	.09*	.16*	-.11*	.08*	-.08*	-.03	.56*	-.59*							
10. Physical symptoms	.03	.08*	.01	.13*	.02	.06*	.40*	-.39*	.52*						
11. Planning	-.01	-.21*	.01	-.02	-.02	-.03	-.20*	.29*	-.20*	-.14*					
12. Marital empathy	-.15*	-.65*	.04*	-.09*	.01	-.04	-.17*	.28*	-.23*	-.15*	.20*				
13. Partner strain	.14*	.48*	-.06*	.06*	-.02	.00	.24*	-.31*	.27*	.23*	-.13*	-.66*			
14. Partner disagreement	.15*	.38*	-.18*	-.02	-.12*	-.08*	.18*	-.23*	.21*	.15*	-.09*	-.45*	.58*		
15. Satisfaction	-.19*	-.57*	.14*	-.06*	.12*	.04*	-.20*	.32*	-.24*	-.14*	.19*	.76*	-.65*	-.47*	
16. Commitment	-.25*	-.49*	.16*	.00	.17*	.06*	-.17*	.23*	-.19*	-.11*	.15*	.60*	-.51*	-.38*	.63*

*Note*: Significant correlations at *p* < .05 are marked with an asterisk.

### Main Analyses

We conducted multilevel regression analysis with the software HLM [41], predicting the odds of divorce over 10 years at T2 based on T1 degree of lack of planning with the partner and various T1 demographic, individual, and relationship variables as controls in four analytic steps. We used multilevel modeling for our analyses because our sample also included data from siblings who are likely to be similar on many of our variables (e.g., positive affect, neuroticism), violating the assumption of independent observations. Thus, we set up our analyses as 2-level models where individuals were nested within families, which controlled for the lack of independence of observations in our analysis by accounting for potential similarities as a result of belonging to the same family of origin. We estimated random intercepts and fixed slopes in our models.

In the first analysis step, we predicted the odds of divorce using only our lack of joint planning with the partner variable. The results of this analysis (see Table 2 for the results of each analysis step) indicated that those who were more likely to plan without their partner had significantly higher odds of divorce (OR = 1.46, *p* < .001) than those who planned together with their partner more.

**Table 2:** Unstandardized Betas, Standard Errors, Odds Ratios, and 95% Confidence Intervals of Predictors in Each Analytic Step.

Predictor	Step 1			Step 2					Step 3				Step 4			
	B	SE	odds ratio	95% CI	B	SE	odds ratio	95% CI	B	SE	odds ratio	95% CI	B	SE	odds ratio	95% CI
Lack of joint planning	.38	.05	1.46	1.33,1.60	.42	.05	1.52	1.37,1.68	.42	.05	1.52	1.37,1.69	.17	.08	1.19	1.03,1.38
Age					-.01	.01	0.99	.97, 1.01	-.01	.01	0.99	.98, 1.01	-.004	.01	1.00	.98, 1.02
Gender					.004	.13	1.00	.78, 1.29	.004	.13	1.00	.78, 1.30	.03	.13	1.04	.80, 1.35
Relationship length					-.06	.01	0.94	.92, .95	-.06	.01	0.94	.92, .95	-.06	.01	0.94	.93, .96
Number of children					.02	.05	1.02	.93, 1.12	.02	.05	1.02	.93, 1.12	.03	.05	1.03	.94, 1.14
Neuroticism									.02	.12	1.02	.80, 1.29	.02	.12	1.02	.80, 1.30
Positive affect									-.02	.13	1.00	.78, 1.28	.11	.13	1.11	.86, 1.44
Negative affect									.21	.15	1.23	.93, 1.64	.18	.15	1.20	.90, 1.61
Physical symptoms									-.03	.09	0.97	.82, 1.15	-.05	.09	0.95	.80, 1.13
Planning									.13	.11	1.14	.92, 1.41	.14	.11	1.15	.93, 1.43
Marital empathy													.16	.20	1.18	.79, 1.76
Partner strain													-.06	.17	0.94	.68, 1.31
Partner disagreement													.31	.11	1.36	1.09,1.70
Satisfaction													-.10	.06	0.90	.81, 1.01
Commitment													-.44	.11	0.64	.51, .81

*Note*: Confidence intervals that do not include zero as a value are significant at p < .05.

In our second analysis step, we predicted the odds of divorce based on lack of joint planning with the partner and the demographic variables (i.e., age, gender, relationship length, number of children). The results indicated that lack of joint planning with the partner was still associated with significantly higher odds of divorce (OR = 1.52, *p* < .001). Gender, participant age, and number of children were not significant predictors of divorce; however, greater relationship length was associated with significantly lower odds of divorce (OR = .94, *p* < .001).

In our third analysis step, we added the individual psychosocial variables (i.e., neuroticism, positive affect, negative affect, physical symptoms, and general tendency to plan) to the multilevel model predicting the odds of divorce. Lack of joint planning with the partner was still associated with significantly higher odds of divorce (OR = 1.52, *p* < .001), whereas relationship length was associated with lower odds of divorce (OR = .94, *p* < .001). None of the other predictors reached statistical significance.

In our fourth and final analysis step, we added the relationship variables (i.e., marital empathy, partner strain, partner disagreement, relationship satisfaction, and commitment) to the multilevel model to examine whether lack of joint planning still predicts the odds of divorce controlling for various indicators of relationship quality. Although the effect was reduced, lack of joint planning with the partner still predicted the odds of divorce (OR = 1.19, *p* = .022), such that each unit of decrease in planning jointly with the partner resulted in a 19% increase in the odds of divorce. In addition, greater relationship length (OR = .94, *p* < .001), higher relationship satisfaction (OR = .90, *p* = .073), and higher commitment (OR = .64, *p* < .001) were associated with significantly lower odds of divorce, whereas greater partner disagreement was associated with higher odds of divorce (OR = 1.36, *p* = .008). None of the other predictors were statistically significant.

In separate analyses, we also examined whether any of the control variables moderated the effects of lack of joint planning with the partner on the odds of divorce. None of the interactions between lack of joint planning and the control variables were statistically significant (*p*-values ranged from .12 to .70). These results indicate that the effects of lower joint planning together with the partner are robust and do not seem to be dependent on the levels of these demographic, individual, and relationship factors, at least in our data. Rather, lack of joint planning predicted higher odds of divorce regardless of individuals’ standing on these variables.

## Discussion

Our findings show that the less people plan jointly with their relationship partner, the more likely they are to experience divorce over time. As the sole predictor, each unit of decrease in joint planning with the partner was associated with a 46% increase in the odds of divorce. It is noteworthy that the increase in odds of divorce was found above and beyond the effects of important demographic, psychosocial, and relationship factors: one unit of decrease in joint planning was still associated with a 19% increase in the odds of divorce. Furthermore, lack of joint planning predicted divorce despite high levels of marital functioning at baseline.

People plan and make decisions jointly with their relationship partner to different degrees. As the associations between relationship quality variables and joint planning indicate, the degree to which people plan jointly with their partner is associated with the quality of their relationship, such that those who are in higher quality relationships are also more likely to plan jointly with their partner. It is likely that to some degree, the quality of the relationship influences people’s desire for joint planning, such that when individuals are generally satisfied with their partner and relationship and there is little conflict between the partners, they are more willing to involve their partner in their planning and decision making. However, given that lack of joint planning predicted the odds of divorce even when controlling for the initial quality of the relationship, the results suggest that the reverse is also true. In other words, a lack of joint planning results in a greater likelihood of relationship dissolution, suggesting that it erodes the quality of the relationship over time. Thus, the association between relationship quality and joint planning is likely to be bidirectional, so that just as worse relationships may lead to less joint planning, less joint planning leads to higher odds of relationship dissolution and thus also likely lower relationship quality.

In addition to individuals’ satisfaction with their relationship and the general quality of the relationship, joint planning is also likely influenced by other relationship dynamics. For example, partners’ level of agreement on various topics (e.g., leisure, finances) and the partner’s responsiveness and understanding would likely influence people’s desire and willingness to plan jointly with their partner. Those who are more compatible with their partner and have fewer areas of disagreement, and those who have partners who show empathy and understanding are probably more willing to plan and make decisions together with their partner. Indeed, joint planning was significantly associated with partner empathy and disagreement between partners. However, lack of joint planning was associated with divorce even beyond these factors, suggesting that joint planning is not solely a function of partners’ shared views on different issues and the responsiveness of the partner. Instead, lack of joint planning independently contributed to the prediction of divorce, even when we accounted for initial levels of disagreement with the partner and the partner’s empathy.

We found similar results with regards to relationship commitment as well. Commitment is a strong predictor of relationship dissolution [33–34], and our study provides further support for the role of commitment in predicting divorce. However, lack of joint planning was still a significant predictor of divorce when accounting for initial levels of relationship commitment. Taken together, given that lack of joint planning was still uniquely predictive of divorce even when we controlled for initial levels of relationship quality and commitment, as well as various factors related to the partners (i.e., disagreement, empathy), our results suggest that joint planning is not just a proxy for a bad quality relationship or incompatible partners, but has important consequences for the relationship over time. More specifically, not considering a partner in planning and decision making can erode a relationship over time and lead to eventual dissolution.

Our findings are in line with the basic tenets of interdependence theory. People’s decisions affect the lives of others and their plans are carried out within the context of close relationships [12–13,23–25,29]. The more interdependent the relationship partners are, such as in the case of married or cohabiting partners, the more one partner’s actions affect the outcomes of the other partner, and the more the partner influences the execution of one’s plans [12–13]. Thus, it is important for relationship partners to try to coordinate their actions in order to reduce the burdens of interdependence and maintain high levels of relationship quality in the face of everyday challenges. Making everyday decisions and long-term plans for the future jointly with the partner likely reduces the difficulties of coordinating actions and preserves the relationship over time. Joint planning likely reduces the potential of conflict between the partners, increases the chances that the partner will be supportive, and makes goal progress easier. Conflict between partners’ goals, lack of partner goal support, and goal progress difficulties in a relationship are all associated with lower relationship satisfaction and well-being [23,25,27].

Although our results show strong support for the important role of lack of joint planning in predicting divorce, given that we only had two time points, we were unable to test the possibility of bidirectional associations between the relationship variables and joint planning. It would have been ideal if we could have shown that controlling for these initial characteristics of the relationship, lack of joint planning predicts changes in some of these relationship dynamics, which then predict divorce. However, we were able to show that controlling for initial levels of relationship quality and commitment, lack of joint planning still uniquely predicted divorce, which is an important step in demonstrating that lack of joint planning is not just a proxy for a bad relationship but has important consequences for relationship dissolution.

Furthermore, given the two time points, we were also unable to test potential mechanisms that would link lack of joint planning to divorce. Lack of joint planning likely makes coordinating the partners’ actions more difficult, and likely results in more conflicts between the partners, less partner support for plans and decisions, and lower levels of goal progress. In existing research, these variables have already been linked to lower levels of relationship quality [23,25,27], but examining their links with joint planning is a potential next step. In future research, it will be important to consider these variables and measure them at multiple time points to explore them as potential mechanisms.

In the current study, we also tried to rule out numerous factors that we thought could explain the effects of lack of joint planning on divorce but our variables did not eliminate the unique effects of joint planning. For example, we controlled for general tendency to plan, partners’ level of disagreement, and partner empathy. However, there are likely other partner and relationship variables that were not assessed in the current study that are likely to be important (e.g., closeness). We would anticipate that joint planning would be associated with most variables that tap into the quality of the relationship, but would continue to predict divorce, given the importance of partners’ ability to coordinate their actions with one another to surmount the challenges posed by living in a highly interdependent relationship.

In future research it will be important to explore the dynamics of relationships with different degrees of joint planning and other potential moderators of the effects of lack of joint planning on divorce. Perhaps joint planning is more important in the case of certain types of issues, and the consequences of different degrees of joint planning might depend on other factors related to the relationship (e.g., how deeply intertwined partners’ lives actually are, how compatible the partners’ goals are). We were unable to answer these questions in the current study, but these issues will be important to examine in future research. Greater understanding of the harms associated with lack of joint planning would increase our knowledge of relationship processes, which could be used to aid couples in dealing with the challenges associated with high levels of interdependence while maintaining high relationship quality over time.
